# Application value of artificial intelligence algorithm-based magnetic resonance multi-sequence imaging in staging diagnosis of cervical cancer

**DOI:** 10.1515/biol-2022-0733

**Published:** 2024-06-11

**Authors:** Rui Chang, Ting Li, Xiaowei Ma

**Affiliations:** Department of Obstetrics and Gynecology, The First Hospital of Yulin, Yulin, 719000, Shaanxi, China; Cancer Diagnosis and Treatment Center, The First Hospital of Yulin, Yulin, 719000, Shaanxi, China; Department of Imaging, The First Hospital of Yulin, Yulin, 719000, Shaanxi, China

**Keywords:** artificial intelligence algorithm, magnetic resonance multi-sequences, cervical cancer staging, feature extraction

## Abstract

The aim of this research is to explore the application value of Deep residual network model (DRN) for deep learning-based multi-sequence magnetic resonance imaging (MRI) in the staging diagnosis of cervical cancer (CC). This research included 90 patients diagnosed with CC between August 2019 and May 2021 at the hospital. After undergoing MRI examination, the clinical staging and surgical pathological staging of patients were conducted. The research then evaluated the results of clinical staging and MRI staging to assess their diagnostic accuracy and correlation. In the staging diagnosis of CC, the feature enhancement layer was added to the DRN model, and the MRI imaging features of CC were used to enhance the image information. The precision, specificity, and sensitivity of the constructed model were analyzed, and then the accuracy of clinical diagnosis staging and MRI staging were compared. As the model constructed DRN in this research was compared with convolutional neural network (CNN) and the classic deep neural network visual geometry group (VGG), the precision was 67.7, 84.9, and 93.6%, respectively. The sensitivity was 70.4, 82.5, and 91.2%, while the specificity was 68.5, 83.8, and 92.2%, respectively. The precision, sensitivity, and specificity of the model were remarkably higher than those of CNN and VGG models (*P* < 0.05). As the clinical staging and MRI staging of CC were compared, the diagnostic accuracy of MRI was 100%, while that of clinical diagnosis was 83.7%, showing a significant difference between them (*P* < 0.05). Multi-sequence MRI under intelligent algorithm had a high diagnostic rate for CC staging, deserving a good clinical application value.

## 1 Introduction

Cervical cancer (CC) is a common malignant tumor of female genital tract with a high incidence [1,2]. With the progress in time, the incidence of CC in younger patients appeared to have been increasing [3]. In 2009, the International Federation of Obstetrics and Gynecology (FIGO) updated the staging of CC. At present, the FIGO staging of CC is mainly clinical staging, without considering the results of surgical pathological examination [4,5]. Clinical staging mainly relies on careful examination by doctors, preferably by experienced doctors, also under anesthesia to facilitate triad diagnosis [6]. According to FIGO clinical staging, when there is any doubt about the staging of a specific tumor, a relatively early stage diagnosis should be made [7,8]. The staging methods of CC include palpation, visual examination, colposcopy, cervical tubation and curettage, cystoscopy, proctoscopy, intravenous urography, and guhe and lung X-ray examination [9,10]. Suspected lesions of the bladder and rectum need to be biopsied and confirmed by histology. Cervical resection or cervical truncation is the diagnosis of micro-invasive carcinoma [11,12]. Clinical staging of CC usually cannot determine the extent of the tumor [13]. During the surgical and pathological staging of CC patients, most of the patients moved to the next stages, and the pelvic and para-aortic lymph node regions were most likely to have occultic metastases [14]. For a long time, gynecological examination has been used clinically for preoperative staging of CC patients, which has certain subjective arbitrariness and low accuracy. In the past, the assisted examination did not rely on the modern imaging, and the error of the examination results was very large.

However, with the increase in the number of patients and the limited medical resources, relying solely on the naked eye observation and diagnosis of doctors directly reduces the medical efficiency and is not conducive to the early detection and treatment of CC. Magnetic resonance imaging (MRI) based on artificial intelligence technology has been extensively accumulated in the diagnosis of CC, and MRI has been determined as the best imaging examination method for the soft tissue and the cumulative range of the uterus in patients with advanced tumors [15,16]. MRI can show different shapes and can be clearly imaged even on a low signal basis because the enhanced scan sequence is not enhanced [17]. The use of MRI has yielded high resolution for the diagnosis of CC; multi-sequence, multi-direction, and multi-parameter inspection will not cause damage to the body. It has good signal sensitivity, can observe abnormal lesions in detail, and effectively reduce the rate of missed diagnosis [18,19]. With the computer-aided diagnosis of image features, the increase in image size does not lead to overfitting. Deep neural network can fully approximate any complex nonlinear relationship, and the residual network avoids the root cause of gradient disappearance. The characteristics of the residual network are easy to optimize, and can improve the accuracy by increasing considerable depth. Its internal residuals use jump links, alleviating the gradient disappearance caused by increasing depth in deep neural networks, and showing good results in the selection of ultra-deep residuals neural networks. The depth residual net method is introduced in multi-sequence MRI to eliminate individual morphological differences and noise interference in the acquired original images, so that images can be standardized to achieve image feature extraction [20]. MRI combined with neuronavigation can update intraoperative images of tumors and functional structures for real-time acquisition. This has a good effect in showing the correlation of the lesions with motor and language-related cortical and white matter fibrin [21]. Deep residual network (DRN) introduces residual connections, allowing the network to retain information from the original input during training, thus solving the common issue of the vanishing gradient problem in deep neural networks. It enables the network to go deeper, reducing training time, and achieving better feature learning, high-performance capabilities, and meaningful feature representation. DRN can learn more interpretable feature representations from data, which is crucial for image analysis, visualization, and interpretation.

Based on this, the research applied the DRN to cancer staging diagnosis based on multi-sequence MRI. By introducing the residual network model and adding feature enhancement layers, the research effectively utilized the feature information from MRI images, thereby improving the accuracy and performance of image analysis. Furthermore, this research investigated the application of deep learning for CC staging, combining multi-sequence MRI for comprehensive analysis, and compared it with other classic deep neural networks. The aim of this research was to demonstrate the good accuracy and sensitivity of using the multi-sequence MRI image analysis with the DRN model for CC staging diagnosis, effectively addressing the clinical staging and diagnostic challenges of CC.

## 2 Methods

### 2.1 Research object

Ninety patients diagnosed with CC from August 2019 to May 2021 in the hospital were included as the research objects. The clinical data and MRI data of these patients were collected. Their age ranged from 24 to 63 years, with 42.6 ± 8.37 years as average; the course of disease lasted for 1 week to 6 years, with an average of (3.6 ± 1.9) years. MRI examinations of all the patients were performed by a gynecologist, and then the clinical staging and surgical pathological staging were performed, respectively.

Inclusion criteria: (1) Inpatients underwent detailed data collection of clinical treatment (including age, gender, previous medication history, and tumor history); (2) combined with imaging data, physical examination, etc., it was examined and diagnosed as glioma; (3) patients who had no other mental illness; (4) they had good understanding and communication skills; (5) patients who actively cooperated with the examination; and (6) cumulative space-occupying lesions were close to functional areas.

Exclusion criteria: (1) Patients who did not agree to participate in this research; (2) the cases with incomplete data; (3) patients who had a heart pacemaker; (4) those who suffered from congenital diseases; (5) those who had the non-titanium metal spinal internal fixation, artificial joints, or orthopedic implant materials; (6) patients who carried various types of biostimulators; (7) patients who received a clipping of intracranial aneurysm; and (8) those who had an implanted cardiac defibrillator *in vivo*.


**Informed consent:** Informed consent has been obtained from all individuals included in this study.
**Ethical approval:** The research related to human use has been complied with all the relevant national regulations, institutional policies and in accordance with the tenets of the Helsinki Declaration, and has been approved by the authors’ institutional review board or equivalent committee.

### 2.2 DRN model

**Figure 1:**
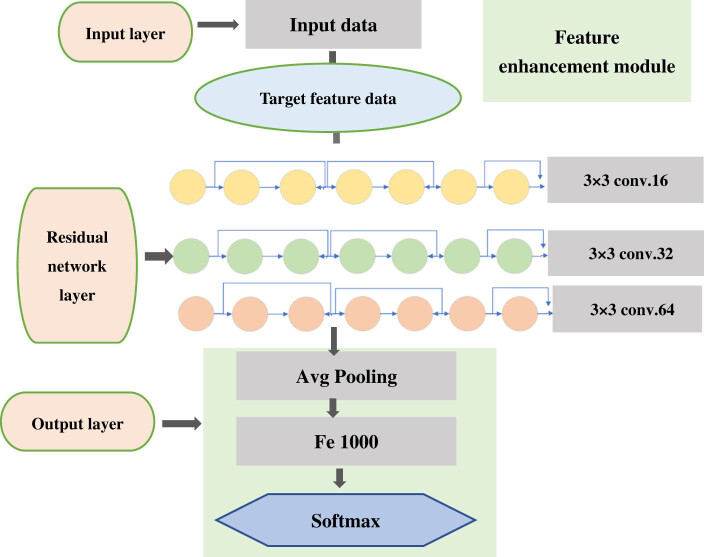
Structure diagram of DRN.

The schematic diagram of the DRN structure is shown in Figure 1. When building the model, the overall parameter adjustment process is relatively important. According to the type of activation function, the types of loss function and weight initialization were determined, and then the network structure was established. The number of hidden layers and the number of neurons in each hidden layer were determined. For the random assignment of hyperparameters, the learning rate was adjusted to obtain a more appropriate threshold of the learning rate, and the initial value was half of the threshold. The number of iterations and the remaining hyperparameters after training were determined through experiments [22]. The detailed hyperparameter settings are illustrated in Table 1.

**Table 1:** Hyperparameter settings

Parameter	Value
Number of network layers	40
Weight initialization	He initialization
Number of neurons	16 × 32 × 64
Learning rate	0.1 (dynamically decreasing)
Activation function	Softmax
Loss function	Cross entropy + Softmax
Regularization	L2 + Dropout (0.5)
Number of iterations	230
Convolution kernel size	3 × 3
Pooling strategy	Average pooling
Amount of data per batch	128

The residual network learning module was composed of identity mapping and residual mapping. When the network reached the optimal configuration, the mapping that deepened the residual of the network would be set to 0, and only the identity mapping relationship would remain. Under the theoretical network, the network would be in the optimal state, the network performance was also deepening, and the residual learning module is expressed as equation (1).

(1) \[{X}_{1}+1={X}_{1}+F({X}_{1},{W}_{1}).]\]

In general, the residual network consisted of more than one residual learning modules. *F*(*X*_1_ *, W*_1_) represented the residual mapping, *X*_1_ stood for the identity mapping, and *W*_1_ was a 1 × 1 convolution operation. This model was used in the case of dimensionality raising or dimensionality reduction. Equation (1) is for a residual learning module, and in the case of a deeper layer *B*, it is expressed as equation (2).

(2) \[{X}_{B}={X}_{1}+\mathop{\sum }\limits_{i=1}^{B-1}F({X}_{i},{W}_{1}).]\]

The layer *B* could represent any layer that was shallower than it and the residual sum between them. The gradient damage function for *X*_1_ is expressed as equation (3).

(3) \[\frac{\partial \varepsilon }{\partial {X}_{1}}=\frac{\partial \varepsilon }{\partial {X}_{B}}\frac{\partial {X}_{B}}{\partial {X}_{1}}=\left(1+\frac{\partial \varepsilon }{\partial {X}_{B}}\mathop{\sum }\limits_{i=1}^{B-1}F({X}_{i},{W}_{1})\right).]\]

The equation could also be expressed as equation (4).

(4) \[\frac{\partial \varepsilon }{\partial {X}_{1}}=\frac{\partial \varepsilon }{\partial {X}_{B}}+\frac{\partial \varepsilon }{\partial {X}_{B}}\frac{\partial }{\partial {X}_{1}}\mathop{\sum }\limits_{i=1}^{B-1}F({X}_{i},{W}_{i}).]\]

Equations (3) and (4) represent two important properties in the residual network.

During the training process, the value of \[\frac{\partial }{\partial {X}_{1}}\mathop{\sum }\limits_{i=1}^{B-1}F({X}_{i},{W}_{i})]\] is always –1, which prevented the disappearance of gradients in the residual network. \[\frac{\partial \varepsilon }{\partial {X}_{1}}]\] meant that the gradient in layer *B* could be directly transferred to any layer that is shallower than it.

The residual learning network is a two-way transmission process, and the feature data information could be propagated between the deep and shallow layers. If *q(X*_1_ *) = a*_1_ *X*_1_, the residual learning module can be expressed as equation (5).

(5) \[{X}_{1}+1={a}_{1}{X}_{1}+F({X}_{1},{W}_{1}).]\]

On the basis of the theory, the unlimited deep layer *B* is expressed as equation (6).

(6) \[{X}_{L}=\left(\mathop{\prod }\limits_{i=1}^{B-1}{a}_{1}\right){X}_{1}+\mathop{\sum }\limits_{i=1}^{B-1}\left(\mathop{\prod }\limits_{i=1}^{B-1}F({x}_{1},{W}_{1}\right).]\]

If only the left side was considered, then the partial differentiation of the damage function is expressed as equation (7).

(7) \[\frac{\partial \varepsilon }{\partial {X}_{1}}=\frac{\partial \varepsilon }{\partial x\text{'}B}\left(\mathop{\prod }\limits_{i=1}^{B-1}{a}_{i}\right).]\]

*a* > 1 in the equation is prone to explosions. When *a* < 1, the gradient table is 0, which would hinder the back propagation of information in the residual network, and would also affect the training of the network. Therefore, *a* here could only be assigned the value of 1, which also indicated that in the residual network, the identity mapping relationship is the optimal choice.

### 2.3 Steps for DRN to diagnose MRI images

(1) Data preparation and preprocessing: a dataset of CC MRI images was collected and acquired, ensuring the quality and suitability of the image data. Preprocessing operations such as image normalization, resizing, and data augmentation were performed to make the data suitable for input to the network.
(2) Network selection and construction: the DRN network model was established, including input layers, convolutional layers, pooling layers, and fully connected layers. Subsequently, the network was customized and modified as needed.
(3) Model training: the prepared CC MRI image data were input into the network for training. Appropriate loss functions (such as cross-entropy loss) and optimizers (such as Adam, SGD, etc.) were employed to update the network’s weights through backpropagation, gradually learning image features and patterns.
(4) Validation and tuning: during the training process, a validation set was set to monitor the model’s performance. Based on the performance on the validation set, the hyperparameters (learning rate, batch size, etc.) or implement strategies such as early stopping were adjusted to improve the generalization ability of the model.
(5) Evaluation and testing: after training was completed, an independent test set was utilized to evaluate the model performance, and metrics such as accuracy, recall, and *F*1 score were calculated to measure the effectiveness of the model in diagnosing CC from MRI images.
(6) Visualization and interpretation: visualization tools were utilized to analyze the attention distribution of the model on the images, understanding the regions the model focuses on during diagnosis. After validation and testing phases were complete, the well-trained model was integrated into medical image analysis software.

### 2.4 Flow chart of CC image preprocessing

**Figure 2:**
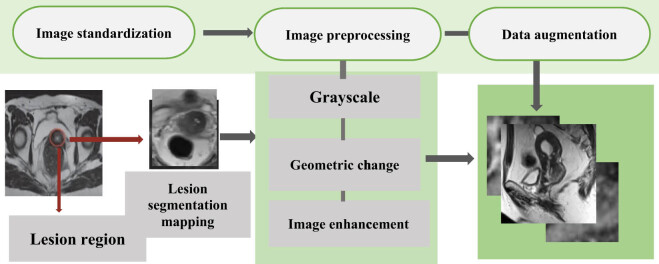
Flow chart of CC image preprocessing.

Deep learning played an important role in the process of medical image recognition. In the whole process of model training, the quality of the number also had a certain impact on the convergence, fitting, and precision of the model [23]. Not all the data images belonged to the same plane layer. In some core regions of the lesions, there was also the situation where the positions of the internal structures differed in the human body, and the feature information presented would also be different. The process of image processing is displayed in Figure 2.

### 2.5 MRI examination

The examination was performed using a Philips Gyroscan NT-1.0T superconducting magnetic resonance instrument in the Netherlands. Superconducting magnetic resonance apparatus was used for the examination.

Before the examination, the patients should properly fill the bladder and be in a supine position.

Repeated communication was made with the patients before the examination, to relieve the patients’ anxiety and nervousness. The matters needing attention during the whole examination process were expounded in details. A detailed and simple training was given to the patients, the precautions needing attention during the examination process were detailed, and some issues during the examination were also told. For example, patients were instructed to close their eyes, minimize swallowing, and relax their minds. There were conducive to promoting the smooth progress of the examination. Body coils were selected. Conventional axial T1 weighted imaging (T1WI) (spin echo, SE) and T2 weighted imaging (T2WI) (turbo spin echo, TSE), as well as sagittal T1WI (TSE) and T2WI fat suppression sequence were performed. The interval between layers was 1.2 mm, and the layer thickness was 4–5 mm, thin-slice field of view was 350 mm, the interval between slices was 0.2–0.6 mm, slice thickness was 1–3 mm, and the matrix was 256 × 169 mm. For T1WI fat, the time of repetition (TR)/time of echo (TE) = 520/15 ms. For T1WI fat-suppression blood flow, TR/TE = 2,100/100 ms, and the time of inversion (TI) = 155 ms. During the examination, enhanced imaging could be performed on the patients, in which after 15 mL of gadolinium-diethylene triamine pentaacetate (Gd-DAPA) was intravenously injected, axial and sagittal T1WI were performed.

The staging standards of FIGO (2000) were adopted. Then, the same experienced physician with at least 5 years of research experience made accurate preoperative staging during the specialist examination of the patients. After the staging was completed, it was compared with clinical staging depending on the analysis results and surgical pathological structures. The diagnostic capability, sensitivity, accuracy, and specificity of CC under the intelligent algorithm were analyzed.

### 2.6 Indicators of performance evaluation

A reasonable evaluation of the performance indicators could effectively evaluate the performance of the algorithm. The image cross-section was evaluated as a two-class issue. The classes predicted by the model and the actual classes were classified into true negative (TN), false positive (FP), true positive (TP), and false negative (FN) (Table 2).

**Table 2:** Sample structure comparison table

Predicted/actual	Positive	Negative
Positive	TP	FP
Negative	FN	TN

The accuracy was calculated using equation (8). The higher the classification accuracy, the better the performance of the algorithm. Precision was the proportion of all predicted positive samples that were truly positive, as shown in equation (9). Specificity, also known as recall rate, represented the proportion of actual positive samples that were predicted to be positive, which was computed using equation (10). When the accuracy and precision were both high, the harmonic average would be higher. If one of them was low, the harmonic average would also be low, and its value would be close to the lower one (equation (11)).

(8) \[\text{Accuracy}\hspace{.5em}=\hspace{.5em}\frac{\text{TP}+\text{FN}}{\text{TP}\hspace{.5em}+\hspace{.5em}\text{FP}+\text{TN}\hspace{.5em}+\hspace{.5em}\text{FN}},]\]

(9) \[\Pr \text{ecision}\hspace{.5em}\text{=}\hspace{.5em}\frac{\text{TP}}{\text{TP}+\text{FP}},]\]

(10) \[\text{Specificity}\hspace{.5em}\text{=}\hspace{.5em}\frac{\text{TN}}{\text{FP}+\text{TN}},]\]

(11) \[\text{Sensitivity}\hspace{.5em}\text{=}\hspace{.5em}\frac{\text{TP}}{\text{TP}+\text{FN}}.]\]

The random-access memory of the hardware platform in the experimental environment reached 128 GB, the graphics processing unit was NVIDIA 1,080Ti × 2, and the central processing unit was Intel Xeon^®^ Silver4110 CPU@2.10 GHz × 32. The operating system was the Ubuntu 16.04. The deep learning framework was PyTorch1. 2.2, and Python 3.6 was selected as the development language.

### 2.7 Statistical methods

All data in this research were collected for the establishment of Excel database, and analyzed in SPSS 19.0. The accuracy, sensitivity, and specificity of clinical and MRI for CC staging were calculated according to the results of postoperative cases. The measurement data were expressed as mean value ± standard deviation (*x̄* ± *s*), while the enumeration data were tested by χ^2^ test and expressed in a percentage (%). Chi-square test was used for counting data, and T-test was used for measurement data. A difference was statistically significant at *P* < 0.05.

## 3 Results

### 3.1 Comparison of different network accuracy rates

**Figure 3:**
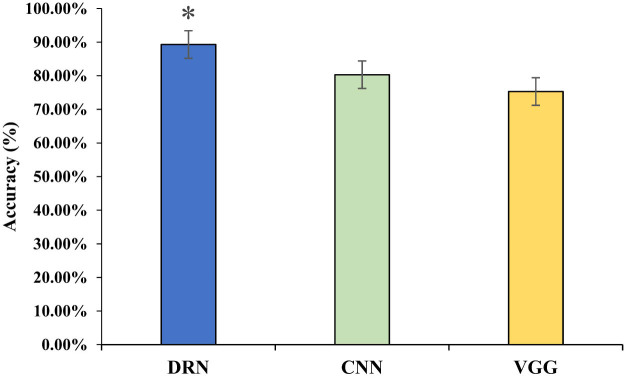
Comparison of different network accuracy rates. (Note: * suggests a great difference with *P* < 005 for comparison between CNN and VGG).

The Cervix Image Database was a specialized database for cervical images, including various types of images such as colposcopic images and MRI images. For the experiment, 10,000 training samples were selected from the dataset. After processing the samples and normalizing the image pixels, all data were divided into several blocks, each containing 100 samples. The analysis compared the recognition rates of different deep learning networks, including the DRN, convolutional neural network (CNN), and visual geometry group (VGG), in processing the images. The results, as shown in Figure 3, indicated that the DRN achieved an accuracy of 89.3%, CNN achieved 80.3%, and VGG achieved 75.3% accuracy in image recognition.

### 3.2 Detection model of DRN

**Figure 4:**
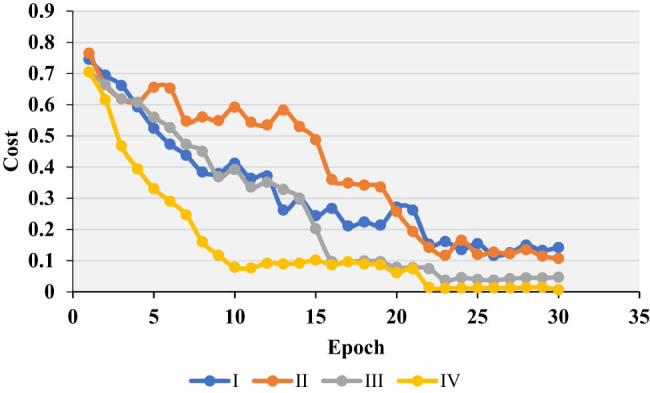
Comparison of weight initialization methods. Ⅰ, Ⅱ, Ⅲ, and Ⅳ were zeroing, randomization, reference [22], and reference [23], respectively.

The weight initialization in the neural network played an important role in the convergence speed of the model and the performance of the network. The neural network training was to adjust the weight parameters, gradient disappearance or gradient guarantee would occur in the gradient descent process. As the four initialization methods were compared, and the results are presented in Figure 4. Then, the initialization method suitable for this research was selected.

### 3.3 Comparison of classification results

**Figure 5:**
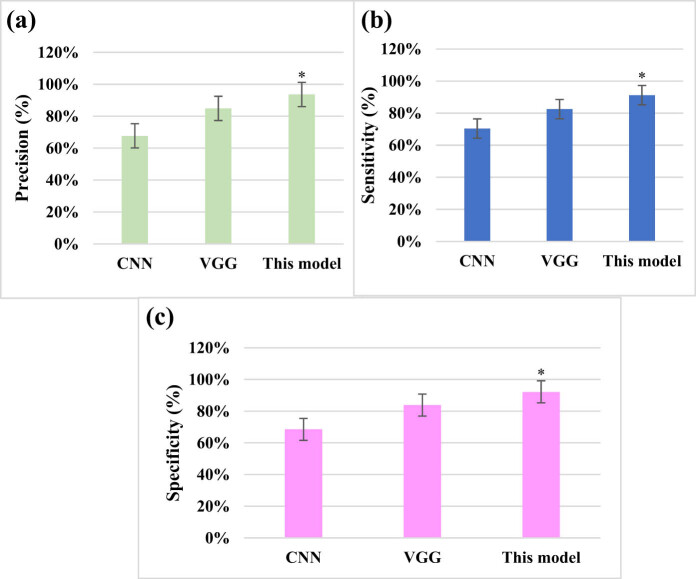
Comparison of model classification results. (a) Precision comparison; (b) sensitivity comparison; and (c) specificity comparison. * Compared with that of CNN and VGG, *P* < 0.05.

The model constructed in this research was compared with CNN and a classic deep neural network VGG. The precision was 67.7, 84.9, and 93.6%, respectively; the sensitivity was 70.4, 82.5, and 91.2%, respectively; and the specificity was 68.5, 83.8, and 92.2%, respectively. The precision, sensitivity, and specificity of the model in this research were notably higher than those of CNN and VGG models (*P* < 0.05) (Figure 5).

### 3.4 MRI images using DRN model

**Figure 6:**
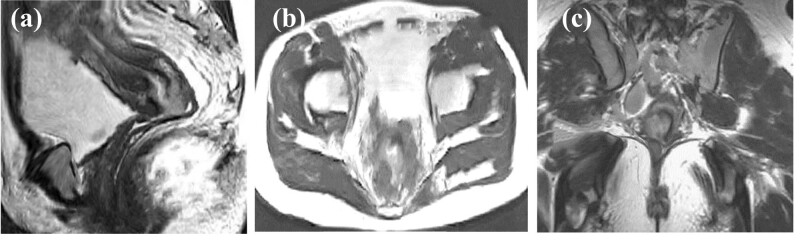
MRI images. (a) Parametrial mass infiltration; (b) enhanced MRI image of the cervix; and (c) parametrial MRI image suggesting high signals on T2WI.

Imaging features of MRI were mainly manifested in cervical enlargement, thickening of cervical mucosa, and complete or partial interruption of cervical interstitium. Enhancement could be observed after enhanced scanning. After enhancement of MRI images, the enhancement was obvious, but was not particularly uniform in the local regions. The tumors broke through the cervix and invaded the upper part of the vagina. After lymph node metastasis, the cervical interstitium disappeared. In some patients, the tumor signal broke through the external serosal surface of the cervix, forming a signal shadow in the parametrial soft tissue, which showed high signals with CC on T2WI (Figure 6).

**Figure 7:**
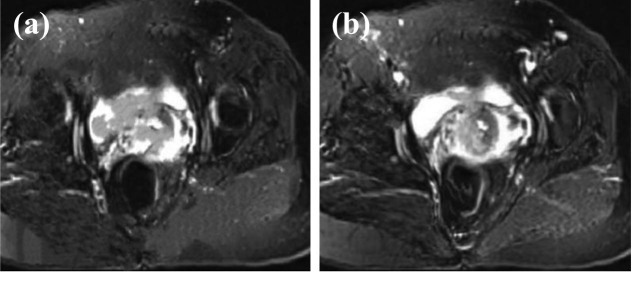
Comparison of MRI images before and after feature enhancement. (a) Original feature and (b) processed feature.

The feature enhancement layer was to highlight the features of the region. It can be observed from Figure 7 that after the feature processing of the highlighted region, the feature region became more complete in Figure 7b. Meanwhile, the effect of enhancement was more obvious. The center region of the feature image after processing could show the integrity of the image more clearly.

### 3.5 Results of diagnostic evaluation

As shown in Table 3, among the 90 patients, the results of stage Ⅰb and stage Ⅱa were consistent between the clinical staging and MRI staging. Five patients showed stage Ⅱb in the clinical staging, but they were classified as stage Ⅱa in MRI staging. Finally, the exploratory surgery was performed for re-examination staging.

**Table 3:** Outcomes of clinical staging and MRI staging

Clinical stage	Number of cases	MRI	Number of cases
Stage 0	0	Stage 0	0
Stage Ⅰa	0	Stage Ⅰa	0
Stage Ⅰb	46	Stage Ⅰb	46
Stage Ⅱa	37	Stage Ⅱa	37
Stage Ⅱb	7	Stage Ⅱb	6

**Figure 8:**
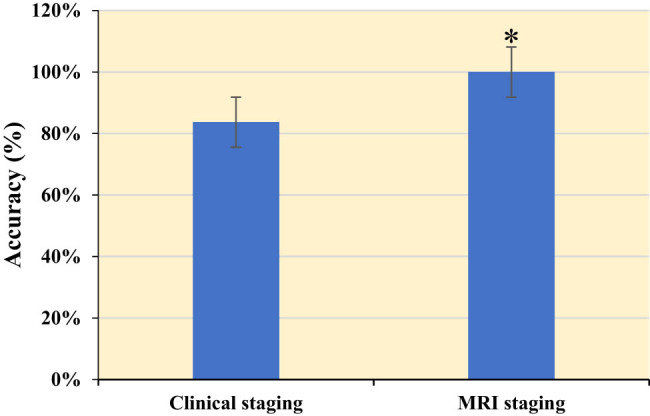
Results of diagnostic evaluation. * Indicated that there was a significant difference between MRI and clinical staging, *P* < 0.05.

In Figure 8, the clinical staging of CC and the MRI of CC staging were compared. The diagnostic accuracy of MRI was 100%, while the clinical diagnostic accuracy was 83.7%, with a significant difference between the two (*P* < 0.05).

## 4 Discussion

The purpose of this work was to investigate the application value of multi-sequence MRI based on intelligent algorithm in the staging diagnosis of CC. A feature enhancement layer was added to the DRN model to establish an MRI image processing method, which was applied to the MRI image processing of CC patients. The structure of DRN allows the network to converge faster, reducing training time. This is highly beneficial for quick training and deploying models in practical applications. DRN also excels in learning hierarchical features from images. Each residual block can capture features at different scales, enabling the network to comprehensively understand the content of the images. DRN has shown outstanding performance in tasks like image classification, segmentation, and object detection [24]. In the imaging domain, DRN can be applied to various areas, including medical image analysis and remote sensing image processing. In this study, we utilized DRN for analyzing CC MRI images, leveraging its capabilities to learn complex features and patterns from multi-sequence MRI data, thus contributing to more accurate and effective CC staging and diagnosis. The accuracy of the model established in this research was 100%. This indicates that multi-sequence MRI of the DRN model has a higher diagnostic rate for CC staging. Due to good soft tissue contrast, MRI is the preferred method for staging local CC, evaluating treatment response, detecting tumor recurrence, and follow-up examination. The doctor obtains diagnostic images, submits characteristic images, and determines the diagnosis. The unique database design structure of intelligent algorithm brings great convenience, and the application of artificial intelligence in diagnostic results is more conducive to reducing diagnostic errors [25]. Liyanage et al. [26] described MRI as an excellent way to describe invasive CC: it can provide an objective measure of tumor size and a high negative predictive value for parastolic invasion and stage IVA disease. Merz et al. [27] demonstrated that MRI is the preferred method for local tumor staging and treatment response assessment to detect tumor recurrence and possible complications. Based on the depth residual network, this research is specially designed to highlight the display effect of enhanced images according to the imaging characteristics of CC images. This model is also identified and analyzed together with other image classification methods. Therefore, this model showed certain advantages. The model provided good staging accuracy on the MRI dataset, which also demonstrated the feasibility of the project.

MRI has important value for CC preoperative staging. It can indicate the presence of enlarged lymph nodes, determine whether lymph nodes have metastasized, and determine the extent of surrounding infiltration. By looking for changes in the cervical signal itself, it can be seen whether there is a tumor and the depth of the cervical invasion. In this way, the anatomical structure of the pelvis can be clearly displayed, and the MRI manifestations and adjacent relationships of various organs can be observed [28]. At present, the clinical treatment principle of CC is based on stage IIb as the dividing line. Before stage IIb, that is, the tumor has not invaded the parastatal tissue, and surgical treatment can be used. After stage IIb, radiotherapy and chemotherapy can be implemented. This also indicates that accurate staging of CC before treatment is conducive to a good choice of treatment. MRI - based imaging diagnosis has incomparable advantages over other imaging examinations. MRI images of patients in this research showed cervical enlargement, cervical mucosa thickening, complete or partial interruption of cervical interstitium, and significant enhancement of cervical area after enhanced scanning. After MRI image enhancement, it can be seen from the image that the enhancement is obvious, but the local area is not particularly uniform. The tumor broke through the cervix to the upper vaginal invasion, lymph node metastasis, and cervical interstitium disappeared. In some patients, tumor signals break through the serous surface of the cervix and form a parastatal soft tissue signal shadow, showing high signal on T2WI as CC. Bourgioti et al. [17] evaluated the predictive power of clinical examination and preoperative MRI for early CC staging. Their results show that MRI is more accurate than clinical examination in evaluating tumors, auxiliary tests, or cervical lesions. Combined with MRI, the predictive power of clinical examination for overall staging (area under curve = 0.59, *P* > 0.05) was significantly improved (area under curve = 0.84, *P* < 0.05). In this research, the clinical staging results of Stages IIa and Ib were consistent with those shown by MRI. In the clinical staging, five cases showed stage IIb, which was classified as stage IIa, and finally modified to exploratory surgery for re-examination staging. The clinical staging of CC was also compared with MRI. The diagnostic accuracy of MRI was 100% and that of clinical diagnosis was 83.7%, the difference was significant (*P* < 0.05). This also proves that intelligent algorithm-based MRI can diagnose the staging of CC and thus better treat the disease.

## 5 Conclusion

The artificial intelligence algorithm was applied to the staging diagnosis of CC in this work. Then, a feature enhancement layer was added to the DRN model to enhance the image information by using the MRI features of CC. Thereout, the model had a high accuracy and specificity. The intelligent algorithm-based multi-sequence MRI gave a 100% diagnostic rate for CC staging, so multi-sequence MRI based on the residual network model in deep learning can be used as a routine examination method for pre-treatment evaluation of CC in staging diagnosis of CC, it has a great clinical application value. There are still some flaws in this research. The specific features of CC staging were not clearly introduced. If the sample size continued to expand, it was unknown whether the constructed model had a better convergence. The follow-up research could continue to investigate from this perspective.
